# A qualitative exploration of the experiences of transdermal alcohol sensor devices amongst alcohol service practitioners (South London, UK)

**DOI:** 10.1080/16066359.2024.2309869

**Published:** 2024-02-06

**Authors:** Eileen Brobbin, Stephen Parkin, Paolo Deluca, Colin Drummond

**Affiliations:** a National Addiction Centre, Institute of Psychiatry, Psychology & Neuroscience, King’s College London, London, UK; b Department of Public Health, Environments and Society at London School of Hygiene and Tropical Medicine, King’s College London, London, UK

**Keywords:** Alcohol, alcohol monitoring, alcohol treatment, technology acceptance model, transdermal alcohol sensor, wearable alcohol biosensor

## Abstract

**Background:**

A qualitative exploration into the views of alcohol service staff on transdermal alcohol sensors (TAS) within three alcohol services in South London, UK. This study aims to assess the acceptability and feasibility of TAS implementation in alcohol services to provide treatment in clinical settings and identify potential challenges and solutions from the perspective of service providers.

**Methods:**

Ten participants, in a patient-facing role with alcohol-related treatment, completed a semi-structured interview.

**Results:**

Three core theoretical themes guided the analysis: perceived usefulness, perceived ease of use and attitudes toward use. Participants thought TAS could be useful as part of alcohol treatment in their service. They thought their service users may face some challenges using the TAS, (such as wearing the device; misplacing it, and/or remembering to remove and replace it for bathing). In general, participant attitudes toward TAS tended to be positive but there were some concerns about the cost and staff training. Participants believed their service users would be skeptical about wearing it, but that it could complement their treatment and motivate them toward their treatment goals.

**Conclusion:**

Results support the acceptability and feasibility of TAS within alcohol services. Participants suggested potential methods of implementing TAS within their treatment plans which could benefit both staff and users. Participants were agreeable and willing to learn more about TAS including the practicalities of implementing TAS. TAS were seen as a potentially useful treatment facilitator, if implemented correctly with sufficiently motivated service users and if specific challenges were addressed.

## HIGHLIGHTS

- The first paper to interview clinical staff from alcohol-treatment services about transdermal alcohol sensors (TAS).
- Staff were positive about the implementation of TAS as a potential adjunct to alcohol treatment.
- Staff were skeptical about applicability and engagement with technology amongst some service users.
- Several practical ways to use TAS were suggested.

## Introduction

Heavy alcohol consumption can lead to alcohol dependence and severe health problems. Specialist alcohol services can provide effective and cost-efficient treatment options when help is needed with alcohol dependence (NICE 2011). Specifically, the staff who work in these specialist alcohol services can use their knowledge, skills, and tools to help those accessing treatment progress toward their intended recovery goals. Transdermal alcohol sensors (TAS) are an innovative technology which can measure alcohol consumption continuously and objectively. This technology, therefore, has the potential to become a tool to facilitate alcohol treatment and intervention used within alcohol services.

As TAS can monitor alcohol consumption continuously, it has been suggested that these could be used to enhance alcohol treatment (Barnett 2015; Dougherty et al. 2015; Wang et al. 2019; 2021). TAS could be used at various stages of treatment and address some of the limitations of the current methods used. For example, a breathalyzer only captures the last few hours of alcohol consumption, or a drinking diary relies on the honesty, engagement, and memory of the individual. There is currently no brand of TAS which is used in clinical settings. SCRAM (Secure Continuous Remote Alcohol Monitoring) is already being used in the criminal justice system in the US and the UK (Kilmer et al. 2013; Bainbridge 2019; Gov.uk 2021; Midgette et al. 2021) and has been demonstrated to be accurate and reliable for monitoring alcohol consumption (Dougherty et al. 2012; Barnett et al. 2014; Alessi et al. 2017; Karns-Wright et al. 2018; Alessi et al. 2019; Fairbairn and Kang 2019). The use of SCRAM in the criminal justice system allows for continuous monitoring of abstinence under a court order or as part of family court proceedings, with consequences for breaching the set terms.

Earlier studies using TAS have been conducted with people who drink socially (Davidson et al. 1997; Ayala et al. 2009; Marques and McKnight 2009; Dougherty et al. 2012; Bond et al. 2014; Hill-Kapturczak et al. 2014; Luczak et al. 2015; Roache et al. 2015; Karns-Wright et al. 2017; 2018; Fairbairn et al. 2019; Fairbairn and Kang 2019; Roache et al. 2019; Croff et al. 2020; Fairbairn et al. 2020; Norman et al. 2020; Rosenberg et al. 2021), people who have been classified as heavy drinkers (Barnett et al. 2011; 2014; 2017; Rash et al. 2019), diagnosed alcohol-dependent individuals (Swift et al. 1992; Sakai et al. 2006; Alessi et al. 2019) and alcohol-related offenders (Goodall et al. 2016). However, no study has explored alcohol service staff perspectives on TAS and their potential utility in alcohol treatment services. Alcohol service staff would be responsible for implementing and using this technology with their service users and are therefore an important omission from existing research. The previous research which used clinical patients, drinking while intoxicated (DWI) offenders or diagnosed alcohol-dependent individuals (Swift et al. 1992; Sakai et al. 2006; Barnett et al. 2011; Alessi et al. 2017; Barnett et al. 2017; Averill et al. 2018; Mathias et al. 2018; Alessi et al. 2019) shows promise for TAS use in clinical treatment settings. These studies have been able to demonstrate TAS accuracy (Swift et al. 1992; Sakai et al. 2006), TAS implementation of CM to reduce alcohol consumption (Barnett et al. 2011; 2017; Averill et al. 2018; Mathias et al. 2018) and that there is a high compliance and TAS return rate (Alessi et al. 2017, 2019). Most of these studies used SCRAM with frequent comments about the size and irritation of the device, so with the development of the newer, smaller, wrist-worn TAS, it will need to be investigated if these meaningful reduce these wearer complaints.

Literature on digital technology and implementation in healthcare in general, suggests that staff factors and other challenges such as service resources, finance, and staff time (Aref-Adib et al. 2019), are important considerations for implementation. Understanding staff and user concerns, beliefs, and attitudes toward such novel technology in the earlier design stages, will help minimize any implementation barriers in alcohol services (Safi et al. 2018). A systematic review of the factors that could help or hinder mobile health (mHealth) adoption found that the two most important technology factors were perceived usefulness and ease of use (Gagnon et al. 2016). The Technology Acceptance Model (TAM) posits that technology use, and usage behavior, are predicted by the perception of the technology’s usefulness and its ease of use (Davis 1985; Chuttur 2009). TAM has been used as a theoretical base to evaluate the implementation of health devices within clinical settings (Holden and Karsh 2010; Portz et al. 2019). While TAS research is a relatively new area of study, identifying facilitators and barriers to development and implementation at the earliest stage has been shown to be important (Gagnon et al. 2012). Therefore, investigation into these potential challenges and potential TAS uses, especially from healthcare staff, is an area of potential qualitative enquiry.

There has been no previous research exploring alcohol staff opinions and experience of TAS use within clinical alcohol settings, that considers whether this technology is appropriate to facilitate alcohol treatment. Responding to this information gap, we undertook an exploratory qualitative interview study to understand views on TAS implementation within alcohol treatment settings. We focused on understanding the acceptance and perception of TAS technology (in the context of alcohol treatment) by clinical staff, in line with the TAM.

The aim of this study is to explore service staff opinion on potential TAS utility within a clinical context, including appearance, ease of use, and potential challenges and solutions to implementation in treatment settings.

## Method

This exploration of staff views on TAS is part of a larger experimental study. In the wider study, service users currently receiving alcohol treatment for alcohol dependence, wore a TAS (BACtrack Skyn) for one week and then completed a semi-structured interview and post-wear survey. However, this article focuses only on staff perspectives on TAS appearance, ease of use and potential challenges of TAS in their clinical setting.

Semi-structured interviews were conducted with staff participants recruited from clinical settings. There were three alcohol services included, all based in south London. Staff were recruited through posters and the researcher attending staff meetings. Participants were currently working as healthcare professionals at one of the participating drug and alcohol services, representing a range of professional backgrounds and roles. All interviews were conducted on MS Teams according to participants’ preferences. Written informed consent was gained for interviews to be audio recorded. This study was approved by the Surrey Research Ethics Committee (Reference: 22/LO/0426).

All staff received a demonstration and explanation of how the TAS works and is worn at the beginning of the interview session.

### Participants

Recruitment occurred between August – October 2022. Participants were required to meet the following inclusion criteria: 1) Health care professional working in an alcohol outpatient or inpatient setting in a participating service, 2) 18 years+, 3) Speak English competently, 4) Able to provide informed consent.

### Procedure

**Figure 1.**
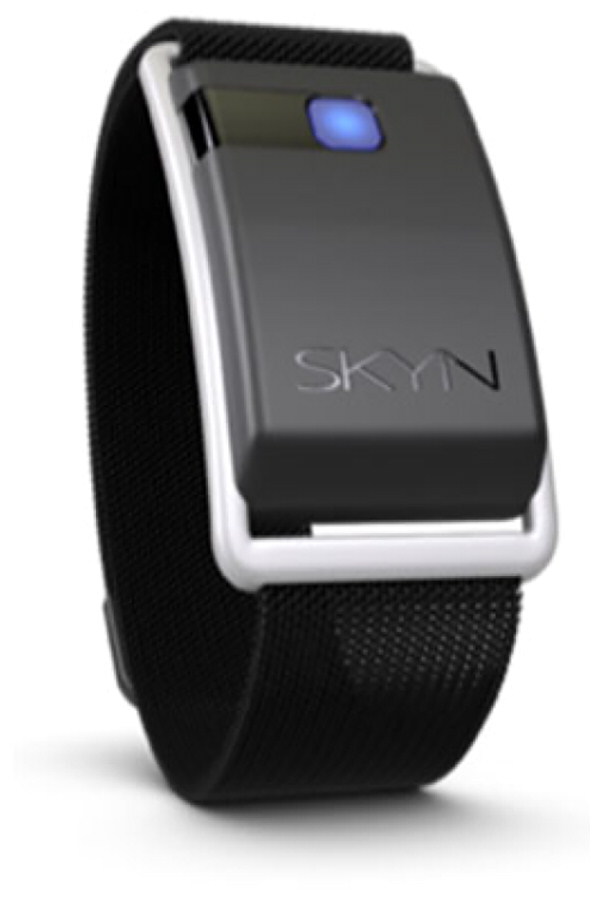
BACtrack Skyn https://skyn.bactrack.com.

All participants provided written informed consent to participate. Participants completed an interview about the implementation of the BACtrack Skyn TAS (Figure 1), and its potential use within alcohol treatment services. At the end of the interview, participants were provided with a £10 shopping voucher to compensate for their time. Interviews were conducted by the same member of the research team for consistency (EB). The audio-recorded interviews were transcribed verbatim by a professional transcription service (attached to King’s College London). All participants agreed to have their interviews audio-recorded and to the use of anonymised quotations in written output. The three components for our topic guide were guided by TAM and concerned: perceived usefulness, perceived ease of use and attitudes toward use.

The researcher who conducted all interviews (EB) had previously led Patient and Public Involvement (PPI) focus groups among staff at the included services and so had already met and built a professional relationship with some participants prior to the interviews. EB has no prior clinical experience of working in alcohol services or any clinical treatment work.

The BACtrack Skyn TAS is worn on the wrist with a sensor in the strap allowing it to be pressed to the underside of the arm. It measures the Transdermal Alcohol Concentration (TAC) at 20-second intervals using this sensor. It also records skin temperature © and motion (g). When worn for personal use the wearer would pair their iPhone using Bluetooth to their Skyn and if the phone is near the TAS it would be syncing the data in near-real time and could be viewed on the BACtrack iPhone Application. For clinical research purposes where the researcher’s phone is paired with multiple BACtrack Skyn, the data is not available to view on the iPhone Application and must be viewed on the BACtrack Skyn server. This limits the ability of real-time viewing. When the data is synced, it will download all the data on the TAS (the last 72 h). New data cannot be viewed until the researcher’s iPhone is next synced to the TAS.

### Analysis

All interview transcripts were uploaded into NVivo 12 (software for qualitative data management and analysis) for coding by which they were categorized into both deductive codes (based on the topic guide and core concepts of TAM) and inductive codes (from themes emerging from the data). A coding framework of the core constructs of TAM was designed by the researcher (EB) and was refined within the research team through discussion in data sessions (Knoblauch 2005) to overcome potential researcher bias. The coding framework was further revised as inductive themes emerged during the analysis. Interviews were transcribed throughout the process and so theme saturation was assessed throughout. The researcher who was conducting the interviews (EB) read and familiarized the transcripts as they were completed and transcribed to start assessing common themes and brought discussions to the research team. Responses relating to each core construct of TAM were further organized into subthemes. The research team met regularly during the coding and analysis process to review data and emerging themes. The main themes that emerged from this process are reported below.

Given the small number of participants, reaching ‘thematic saturation’ of data was not considered an analytical issue as the authors concur with Braun and Clarke (Braun and Clarke 2021), that ‘meaning is generated through interpretation of data … and therefore judgements about ‘how many’ data items and when to stop data collection, are inescapably situated and subjective, and cannot be determined in advance of analysis’.

## Results

### Participant characteristics

A total of 10 participants took part and completed the study (Table 1). There was a mix of male and female participants with an average age of 49 years. While four participants had heard of TAS before, none had used one themselves. The main themes of the analysis are shown in Table 2 below.

**Table 1.** Participant data.

Characteristics	Male (*n* = 4)	Female (*n* = 6)	Combined (*N* = 10)
Age: Mean	53	46	49
Ethnicity	4 White British	2 White British, 1 White Italian, 1 Black Caribbean, 1 Indian Caribbean, 1 Mixed British	6 White British, 1 White Italian, 1 Black Caribbean, 1 Indian Caribbean, 1 Mixed British
Job title	3 Assertive outreach alcohol practitioners 3 Recovery workers 1 Consultant lead in Addiction 1 Clinical Psychologist 1 Substance misuse nurse 1 Smoking cessation advisor		
Heard of TAS	*N* = 2	*n* = 2	*n* = 4
Experience with TAS	*N* = 0	*n* = 0	*n* = 0

**Table 2.** Key themes from interviews.

Themes
Perceived ease of use of the BACtrack Skyn
Challenges
Perceived usefulness of the BACtrack Skyn within alcohol treatment
Benefits of the BACtrack Skyn from a staff perspective
Service user motivation
Attitudes toward using the BACtrack Skyn
Device appearance
* TAS compared to other methods of alcohol measurements*
Thoughts on service user acceptance and attitude toward the BACtrack Skyn

## Key themes from interviews

The key themes emerging from the analysis of the interviews are shown in Table 2.

### Perceived ease of use of the BACtrack Skyn

Participants described how they believed TAS could be useful or beneficial within alcohol treatment. While no participant had personal experience of using a TAS, they were asked about their willingness and how they perceived TAS potential ease of use. We recognize this should be further investigated in the future after staff have used TAS themselves with clients.

Responses showed an openness and willingness to learn more. For example, this can be seen in the following statements by Participants 1, 7, and 9 respectively:

From my experience across most addiction services, where there is any sort of device or intervention that is helpful for a client… it can be acceptable. [Male, Assertive outreach alcohol practitioner].

It’s something that’s worth looking at. [Male, Recovery worker].

I think we are always looking for better ways to support our clients.[Female, Substance misuse nurse].

However, there was also shared agreement about concern regarding training and device maintenance. For example, Participant 1 made the following comment:

…it’s about time management and the time it would take for each individual member of staff to do that for each client. Once they’re up and running I’m pretty sure that it would be quite easy to use… [Male, Assertive outreach alcohol practitioner].

They were also asked what they did not like about the TAS and the challenges they foresaw. These challenges are summarized in the next section.

### Challenges

A range of challenges of TAS use in clinical settings were reported (Table 3, below). These were grouped into three main categories: Engagement, Functional, and Population challenges, each of these with its own subcategories of self-reported, expected, challenges.

**Table 3.** Staff challenges.

Challenge category	Sub-categories	Challenge	Quote example
Engagement	Service user engagement	Some service users struggle with engagement, it might be challenging to have them to engage long enough to obtain meaningful data	It engages clients that are willing to engage. Some clients really struggle with engagement and following guidelines. Even if they agree initially, will they remember to take it off when they’re showering… put it back on… will they engage long enough for meaningful detail… I’m not sure if it would work for everyone. That doesn’t mean that it wouldn’t work… because I think it could. Participant 4. [Female, Assertive outreach alcohol practitioner].
		Some staff were not sure if service users would understand the benefits of using the TAS	If clients don’t understand the importance of wearing it, and the importance of charging it and keeping it charged, then that can be a barrier. Participant 4. [Female, Assertive outreach alcohol practitioner].
	Staff engagement	Staff absences. If there is one main person trained to use the TAS within the alcohol service it would be a challenge if they were unavailable (e.g. on leave, sick leave)	If a member of staff was the main person to support a client with starting to use a TAS, and they’re unfortunately unavailable… You would need two to three members of staff fully trained in using them so they could take over. That would be one of the challenges, to ensure there’s enough members of staff for each service to cover. Participant 1. [Male, Assertive outreach alcohol practitioner].
		The frequency of visits required for downloading the data would be an extra burden	Given our caseloads, I’m not sure the practicality of it, especially if you have more than two [using a TAS]. That might be tricky in terms of working. Participant 4. [Female, Assertive outreach alcohol practitioner]. It’s not sustainable to do weeklies. Participant 7. [Male, Recovery worker].
	Regular data downloads	Usually see service users once a week or once a fortnight so would be challenging to complete regular data downloads (multiple times per week)	If you had to see quite a few clients more than once a week that may be problematic. Participant 4. [Female, Assertive outreach alcohol practitioner]. If the information from the device can be checked once a week, then it would be much easier, so to reduce the frequency of clients coming in and see us. Participant 5. [Female, Consultant lead in Addiction]. That 72 h would be very tricky. Participant 7. [Male, Recovery worker].
		Many service users don’t own an iPhone so can’t complete the data downloads	If somebody has that technology and that can be collected automatically that would be useful. Participant 2. [Male, Assertive outreach alcohol practitioner]. It would be more beneficial if they could [sync to Android]. Not everybody likes iPhones… think about all the thousands of people who’ve got phones that are not iPhones. Participant 6. [Female, Recovery worker].
		A challenge for service users attending regular meetings	The client coming in every 72 h for us to deal with the data, we don’t have that time, they don’t have that time necessarily, so that would be a problem. Participant 2. [Male, Assertive outreach alcohol practitioner].
	Service user forgetfulness	Forgetting to charge the TAS	Especially if they’re drinking heavily, they might forget to charge, they might misplace the wristwatch [TAS]. Participant 5. [Female, Consultant lead in Addiction].
		Forgetting to remove/replace before and after showers	I think the only issues we would find are… they would forget to use it or charge it, or possibly go into a shower with it. Participant 1. [Male, Assertive outreach alcohol practitioner].
		Misplacing or losing the TAS	They might forget to charge, they might misplace the wristwatch, might lose it. Participant 5. [Female, Consultant lead in Addiction].
	Removing the device	Service users might remove the TAS when drinking to hide alcohol consumption (tampering)	My only concern is knowing our clientele, if they do relapse, they will either take it off or somehow try to skew the results by doing something, because they don’t want to be seen as failing. I think if there’s an incongruence between the device and they’re still drinking… some clients may take it off. Participant 4. [Female, Assertive outreach alcohol practitioner].
		TAS loss or damage	People losing them, throwing them away, breaking them, people just refusing to be part of it. Participant 2. [Male, Assertive outreach alcohol practitioner]. We give people mobile phones, or used to, just cheap things, and so many just get lost within days, and they’re not even worth pawning, but they’ll try. Participant 2. [Male, Assertive outreach alcohol practitioner].
Functional	Cost	Cost of the TAS, insurance and alcohol service budgets	I don’t know how much they cost, so you’d have to look at budgeting. Participant 6. [Female, Recovery worker]. I’ve no idea of how much the costs are of them, so I would look from the insurance kind of side. Participant 8. [Female, Recovery worker].
		Cost and resources of providing an iPhone to pair with the TAS for staff and/or users	If they haven’t [got an iPhone] and they’ve only got the Samsung, it’d be left to us to do, in the center. And then the service provides Android, so it’s not ideal either… It would be more beneficial if they could [work with Androids too]. Participant 6. [Female, Recovery worker].
	Waterproof	Forgetting to remove when bathing and causing water damage	Will they remember to take it off when they’re showering, and will they remember that when they’ve finished showering to put it back on? Participant 4. [Female, Assertive outreach alcohol practitioner].
Population	Age	Staff were unsure if older individuals would entertain the thought of wearing a piece of technology	I guess maybe even the older generation, as the gentleman the other day wouldn’t even entertain the thought of it, I mean I’m homogenizing, but I think older clients might be a bit wondering what the hell it’s all about. Participant 2. [Male, Assertive outreach alcohol practitioner].
	High risk drinking	Staff wouldn’t consider using it with high-risk drinkers as they would not want to add another responsibility to their treatment	I don’t know how well this would be suitable for a heavy drinker. Participant 7. [Male, Recovery worker].
	Stigma	Service users may not want to wear a TAS continuously for weeks or months at a time due to stigma	Especially people who don’t like interaction, or are busy at work, they feel the embarrassment and stigma of it. Participant 6. [Female, Recovery worker]. People, still functioning and still working, it might be a little bit of a barrier for them. Participant 7. [Male, Recovery worker].

Within Engagement, there were five sub-categories: service user engagement, staff engagement, regular data downloads, service user forgetfulness and removing the device. All of these are linked to the staff and service users engaging with TAS use. These sub-categories included challenges to do with engaging with wearing the TAS and the risk of removals, forgetting to replace it, losing the TAS, meeting regularly to download the data before being over-written and TAS damage.

Currently, the BACtrack Skyn requires syncing to an up-to-date iPhone every 72 h for data download and staff presented this as an important barrier. The staff themselves may not be able to meet this regularly to download the data, service users may not be able to meet regularly, and many do not own an iPhone to complete data download themselves. The BACtrack Skyn is also not waterproof meaning that participants may forget to remove it and shower with it, or if they do remember to take it off, they may not remember to replace it, thereby not providing accurate data. These views were expressed by every member of staff participating in this study.

The Functional category covered sub-themes related to the device itself, including cost and water resistance. Staff were concerned with the cost of TAS itself and how much it would be to purchase and insure them but also with staff resources and time and additional costs of providing iPhones to TAS wearers. A further concern was the TAS not being waterproof and this potentially led to water damage if wearers forgot to remove it.

The Population category includes age, high-risk drinking and stigma related to this specific group of alcohol-dependent individuals accessing alcohol treatment. These sub-categories would not apply to all service users, but staff felt that older individuals may not entertain the thought of using and wearing technology and that some may not want to wear it due to perceived stigma. Some staff also described that they would be unlikely to use TAS with someone who currently drinking heavily as they would not want to burden them with an additional and unnecessary responsibility.

### Perceived usefulness of the BACtrack Skyn within alcohol treatment

In the interviews, participants expressed positivity to TAS use as part of alcohol treatment. As this asked for their thoughts and willingness to use it only and did not ask them to physically use it, participants did emphasize that these benefits and suggestions would need to be tested when staff could use TAS themselves, for example (Participant 1):

I think in terms of alcohol treatment, having a device like that would be helpful and supportive for the clients… we would need to see the outcome of the data to see how useful it would be. [Male, Assertive outreach alcohol practitioner].

### Benefits of the BACtrack Skyn from a staff perspective

The key benefits in clinical treatment were perceived to be its objective alcohol monitoring and use as a motivational tool (such as motivating service users to track their alcohol consumption and reduction where necessary). TAS monitoring allows for an accurate and detailed visual for both staff and the wearer (a visually documented record of alcohol consumption as a spreadsheet which can be printed out or graphed). The reason for TAS use, the length of time of wearing the TAS, and how they use the data, will be different for everyone. An additional benefit for staff is that the TAS places the responsibility on the service user, they would be responsible for wearing and charging the TAS. This placed the responsibility on the service user for maintaining and operating the TAS, rather than the member of staff.

TAS are objective and therefore can be used to observe alcohol consumption between meetings. This would allow a document that could be printed out and seen by the key worker and service user to discuss progress. One participant suggested using TAS to aid in alcohol reduction. The data output could be used to track and aim for reduction starting with goals of reducing peak alcohol consumption or recording half days of abstinence. When discussing the usefulness of TAS during different stages of treatment, one participant commented:

Absolutely, I think that’s a great idea. A lot of our clients get very anxious doing a reduction programme, and lots of different professionals have slightly different ideas on what’s safe reduction, so actually if … they could monitor themselves on their phones, they’ve had this amount, this day and this amount a week later, that could be really good. **Participant 2.** [Male, Assertive outreach alcohol practitioner].

Similarly, when asked about TAS benefits, another stated:

[One] I could think of clients who are wanting to reduce, it gives them sort of a positive reinforcement when they can actually see when it’s actually downloaded, and we can show them the progress they’ve made. Two, it means that it’s user-friendly, we don’t need to have to walk around with a breathalyser test. I don’t get somewhere and think ‘Oh gosh, I’ve forgotten the breathalyser test!’ It places the onus on the client to wear it, not involve much involvement from me. **Participant 4.** [Female, Assertive outreach alcohol practitioner].

### Service user motivation

Staff felt an important factor for the TAS being effective would depend on the user’s motivation. TAS use would be voluntary, the service user must not only consent to wear the TAS but be motivated to keep wearing and using it for it to be effective. They must be motivated to wear it and then the TAS can work as a motivational tool to help them progress with treatment goals. It could also be used to demonstrate the service user’s motivation to engage in wider treatment activity. These views may be noted in the following:

It could be a good tool to be used as a motivation factor for funded treatment. Say look, you want a detox, you want rehab, this requires … ask(ing) people to attend active groups as part of motivational commitment on their part, and it might form a good part of that package of commitment, wearing the watch [TAS], so it then shows about engagement. **Participant 3.** [Male, Smoking cessation advisor].

It engages clients that are willing to engage. Some clients really struggle with engagement and following guidelines. Even if they agree initially, will they remember to take it off when they’re showering… put it back on… will they engage long enough for meaningful detail… I’m not sure if it would work for everyone. That doesn’t mean that it wouldn’t work… because I think it could. **Participant 4.** [Female, Assertive outreach alcohol practitioner].

### Attitudes toward using the BACtrack Skyn

Attitudes were divided into three different sections: 1) Attitudes toward the BACtrack Skyn appearance, 2) attitudes toward the BACtrack Skyn in comparison to the current alcohol monitoring methods they use with service users, and finally 3) staff perspective on service user attitudes toward TAS use as part of alcohol treatment.

#### Device appearance

Participants thought the Skyn appearance had improved considerably from previous versions of TAS, often comparing it to a fitness monitor or a watch (see below interview extract). They described the TAS as ‘sleek’, ‘discreet’ and ‘modern’ and stated from appearance alone, that it was not an obvious device for monitoring alcohol use. For example:

It looked like a Fitbit, so it’s like unimpressionable to the eye, if you know what I mean? People wouldn’t be drawn to it and go ‘ooh, what’s that?’ It looks quite like a smartwatch, so that’s cool. **Participant 6.** [Female, Recovery worker].

#### TAS compared to other methods of alcohol measurements

Current methods commonly used in alcohol services include breathalyzers and self-report diaries. Participants varied in how often they reported using a breathalyzer. The main difference they noted between a breathalyzer and a TAS was that the breathalyzer can only capture a moment in time while the TAS can monitor continuously. Due to this, TAS could be used as a motivational tool to track progress. It could be used to support treatment goals of reduction or abstinence by providing a record of consumption. A breathalyzer is unable to record a reduction in alcohol consumption each week.

Having learnt a little bit about it, it is useful for the client to be able to monitor their own use, but also knowing that I will be able to see that data, which might give them a little bit more motivation to continue with that period of abstinence. **Participant 2.** *[Male, Assertive outreach alcohol practitioner].*

It means that more onus is placed on the client to wear the TAS, as opposed for us to, especially doing home visits, having to remember the breathalyser and to remember to calibrate it and all these other things that we have to do, it’s just easier if the client is wearing the TAS and wearing it appropriately, it’s less work for us, easier to monitor, more user friendly for us. **Participant 4.** [Female, Assertive outreach alcohol practitioner].

If they had the TAS at the beginning you’ve got it there and then, you don’t have to ask them, then you can work with what you’ve got [alcohol consumption levels]. **Participant 6.** [Female, Recovery worker].

But I think a TAS would give you more of a chance [to get accurate data] than a drink diary. **Participant 7.** *[Male, Recovery worker].*

Another difference is that TAS places more responsibility on the TAS wearer than on the staff member. The staff member is responsible for training and learning how to use a breathalyzer, taking it with them to home visits or finding it for service meetings, and remembering to keep it calibrated before they use it each time. However, Skyn would be the responsibility of the service user to wear and charge. For example:

But obviously it depends on the client, I think. But I could see it really working well with clients, especially if you want to show progress over time, to use a device like this. **Participant 4.** [Female, Assertive outreach alcohol practitioner].

Staff noted there are factors that can interfere with accurate alcohol reporting. These include cognitive impairment (relating to memory), language difficulties, and/or limited literacy/numeracy skills. When drinking heavily, it is difficult for the individual to accurately track alcohol consumption. Therefore, staff noted that many service users do not return completed drinking diaries. The difference with the Skyn device is that the TAS removes the service user requirement to remember consumption as the device automatically captures it. Instead, the service user must remember to remove and then replace after showers.

#### Thoughts on service user acceptance and attitude toward the BACtrack Skyn

Participants said that Skyn would need to be considered case-by-case and would depend upon personal choice and the stage of treatment. In general, they thought service users may be skeptical of being monitored but that some would feel positive and enthusiastic about wearing it. If reducing their alcohol consumption is something that they are motivated to do, participants thought the option to use TAS would be beneficial. They did not think that TAS would undermine the relationship between the service user and key worker. These views may be noted in the following:

I think initially they’ll be quite sceptical, quite suspicious. They may not want to be monitored, and they may think that they could be persecuted. I think they might have some negative thoughts about it, especially if someone is continuing to drink or maybe not telling us the whole truth. Whereas certain clients who are very proud of their abstinence would be more than happy to be part of that. **Participant 2.** [Male, Assertive outreach alcohol practitioner].

If they’re in that place where they want to make changes, and they feel it could benefit them to self-monitor a little bit, then I don’t think that would be undermining their trust. **Participant 7.** [Male, Recovery worker].

### Solutions to potential barriers of TAS use in alcohol services

Our secondary objective of this exploratory work with staff participants was to identify potential barriers and facilitators to the implementation of TAS within alcohol services. Participants discussed these challenges and self-reported solutions from their perspective as service providers. The challenges were included in the perceived ease of use section above (Table 3) and here we consider the self-reported solutions staff suggested (Table 4).

**Table 4.** Staff solutions.

Sub-category	Solution	Quotes
Staff engagement	Longer storage capacity so data download meetings don’t need to be as regular	If you can increase that 72 h. Participant 7. [Male, Recovery worker]. A training group for the staff would be useful, comprehensive manual, as well as a YouTube video or a video of some kind from the manufacturer about the uses and a comprehensive list of FAQs that you can become familiar with that most likely a client would ask, so you’ve got the full idea of how it works to take it to a client. Participant 3. [Male, Smoking cessation advisor].
Regular data downloads	Longer storage capacity, being able to download the data on phones other than an iPhone. This could mean the service user could complete the data download themselves	The most I see them is once a week, and mostly fortnight… So it would be a lot of more work physically, and I think that would definitely be an issue. Participant 10. [Female, Substance misuse nurse].
	Being able to pair them with phones other than an iPhone	It would be brilliant if the person could sync it to an app on their own phone and see it, but then obviously that would sync with yours without having to physically meet. Participant 9. [Female, Clinical psychologist in Addiction].
	Longer storage capacity so data downloads don’t need to be as regular	I think the improvement is about the length of time it can hold the data, so that you can reduce the number of times you can see the client within a week. Participant 1. [Male, Assertive outreach alcohol practitioner].
Forgetfulness	Making it waterproof	If it [BACtrack Skyn] was waterproof that would be amazing… There’s a very high risk of memory loss. So, for accidental purposes if it could be waterproof. Participant 8. [Female, Recovery worker].
	Provide service users with a treatment leaflet	I find that if you sit with people and you take them through it, and you make sure they understand, and to show you an example, I think they’re more likely to continue with the use and the confidence of having it. Participant 3. [Male, Smoking cessation advisor].
Stigma	Alcohol dependency can cause embarrassment and stigma for the individual, but these TAS look like a health watch so could be worn without embarrassment at work/in public and allow someone to monitor their alcohol without feeling worried about judgment. They could be further personalized by being able to choose different colors or straps depending on the individual’s preference	[It looks] …a little bit of a look like some sort of legal police device that they use for tracking you. But it also looks a bit like a fitness band, very sleek. If you can personalize it, different straps like a Swatch Watch, because it’s a bit black and a bit standy-outy on the wrist, so if it was yellow or red it may look more like a fashion item. And people like to personalize their things. Participant 2. [Male, Assertive outreach alcohol practitioner].
Cost	Being able to pair other phone brands with the TAS to remove need of service users owning an iPhone	I think the iPhone thing is really tricky. I think if we had a magic wand, that the device could sync with non-iPhone devices. Participant 9. [Female, Clinical psychologist in Addiction].
Technology	If they had the technology to complete the data downloads themselves rather than the staff. If they could pair the device with a non-iPhone (the phone they already bought and own) or a ‘home dock’.	Maybe that they don’t need an app, but there’s a home dock that comes and just syncs it, and then when you see them you can then review the data, rather than it having to be an iPhone that it syncs with. Participant 9. [Female, Clinical psychologist in Addiction].
	Being able to use their own phone to see the device data being collected	Memory should be quite longer, at least a week or two weeks, if it can hold that information, or even download to their phone and then when we come, we can access it or something like that. Participant 10. [Female, Substance misuse nurse].
	Being able to collect other data like sleep or heart rate	A lot of the current smartwatches capture heart rate and sleep, people often describe the difficulties with sleep, be it related to alcohol or not. Participant 3. [Male, Smoking cessation advisor]. It would be brilliant to see sleep rate and unit conversion. Participant 8. [Female, Recovery worker].
	Linked with an app that could convert the units/amount of alcohol being consumed or calories or units to see the data on their smartphone or to input other feedback such as mood	Having an interactive system to input information about how you feel, maybe they can click on some icons and say I feel great today, I feel irritable, I feel anxious, this kind of information on emotions might be quite useful… as well as then the encouraging messages about you’ve reduced by X amount. Participant 5. [Female, Consultant lead in Addiction].
Waterproof	If waterproof wouldn’t need to remember to remove it	In the future, if they made waterproof, that may be better. Participant 4. [Female, Assertive outreach alcohol practitioner]. I think the waterproof thing would be really beneficial. Participant 8. [Female, Recovery worker].

The sub-categories included in this table are: Staff engagement, regular data downloads, forgetfulness, stigma, cost, technology and waterproof. These solutions are ones that the staff self-reported in response to the challenges they saw with TAS implementation in services. When thinking of solutions staff were not bound by limited resources, finances, or time.

A recurring theme throughout these different sub-categories is the solution to increase data storage or make the data downloads compatible with any type of phone. This can be seen in staff engagement, regular data downloads, cost, and technology. Time and access to data are the main barriers to staff and by removing this difficulty the increase in ease of use for staff would be great. The challenge of 72-h storage capacity is removed if wearers could sync with their current phone rather than requiring an iPhone so they could complete the download themselves and the staff could see data in near real-time. This solution would also help with the cost of implementing TAS by removing the requirement for up-to-date iPhones.

Other solutions or suggestions to increase TAS usability to staff were to make it waterproof to reduce the risk of damage, being able to personalize it with colors which may reduce stigma by making it more fashionable and increase the type of data it collects to include physiological and mood data could be useful for the staff and in feedback to the wearer.

There were no solutions self-reported by staff to resolve the lack of service user engagement. Staff did emphasize that involvement would be voluntary and consensual. It would be the responsibility of the staff to provide the information on the TAS and then, if interested, service users could be willing to try it. A participant commented:

And because it’s optional, if someone doesn’t want to use it then of course that’s fine too. **Participant 2.** *[Male, Assertive outreach alcohol practitioner].*

## Discussion

TAS are relatively novel technology, and research into their potential use is still developing. Aside from one brand, SCRAM, there is limited real-world use of these devices thus far. We believe, this is the first study to interview service staff on this topic and therefore provides important knowledge on TAS use within alcohol treatment. No previous publication has used this interview design with alcohol service staff in TAS research. There has been limited research in clinical settings with TAS. The previous studies that have included alcohol-dependent diagnosed individuals or targeted alcohol reduction do suggest there is acceptability and feasibility in the use of TAS in alcohol treatment (Barnett et al. 2011; Dougherty et al. 2014; 2015; Alessi et al. 2017; Barnett et al. 2017). We designed an interview study to ask staff their views on this topic, whether they would be willing to use TAS, how they think TAS could be used and received by service users and their opinion on the BACtrack Skyn specifically. The contributions of staff can provide a greater understanding of previous research regarding TAS clinical use by providing another perspective that is different to a research group (Barnett 2015; Goodall et al. 2016; Caluzzi et al. 2019; Wang et al. 2019; 2021). The findings show support for TAS acceptability within alcohol services from a staff perspective. Participants were positive about Skyn clinical use and appearance, and they believed that implementation in the context of alcohol treatment would be feasible if certain challenges were suitably addressed. Although they were skeptical of how service users would perceive and engage with the TAS.

Staff felt there were many uses for the TAS at different stages of treatment, but the main benefit was the ability to measure alcohol consumption objectively and consistently. TAS allows for a greater amount of detailed information than a breathalyzer or self-report could reliably or routinely provide. They endorsed TAS use in combination with alcohol treatment methods: 1) It could be used when individuals first present at the service to determine alcohol consumption before detoxification. 2) It could be used to aid daily alcohol measurements during a community alcohol service detoxification. 3) It could be used to provide a way to track and confirm alcohol abstinence or reduction to provide personal motivation for the service user in achieving their personal treatment goals. All these take advantage of the continuous monitoring ability of TAS, that current methods cannot. Staff frequently articulated a belief that the TAS would provide a user-friendly and less resource-intensive tool for alcohol measurement compared to the current breathalyzers and self-report drinking diaries, in addition to being the responsibility more of the service user than of the staff. However, staff were also concerned about challenges with TAS loss, damage, tampering and data collection.

During the interviews, participants were asked what challenges they foresaw working with TAS in alcohol treatment. Challenges identified broadly fit into two categories: those that are to do with the TAS itself and those that may be faced by the staff administering or service users wearing the TAS.

The challenges due to the TAS itself have limited solutions that could be implemented by staff. Device cost, technology and data output would require hardware changes from the TAS manufacturer or service funding. These challenges demonstrate that TAS effectiveness itself is not the only influencing factor for implementation, from the staff perspective.

The second category of challenges involves the practical daily use of TAS. These may be able to be addressed by implementing appropriate solutions. Still, it is most likely that the suggestions would involve at a minimum staff time, if not other valuable resources. Other studies have also alluded to these potential challenges with the alcohol-dependent population and so there seems to be an agreement in the awareness of daily wear challenges (Barnett et al. 2011; Leffingwell et al. 2013; Alessi et al. 2017; Mun et al. 2021). Currently used methods, such as breathalyzers, also have time and resource burdens, for example, calibration, and remembering all equipment (enough mouthpieces). The ability to monitor 24/7 with the TAS will have to be weighed up against its technology and time burdens (remembering to charge the TAS/pairing phone, time to learn how to use it, time between downloads) and if any of the mentioned barriers could be reduced in future TAS generations. One staff mentioned the additional time and resources it would take to set up the use of TAS in services, however, once this set up time had been put in place, they then should increase ease of use.

A key theme that appeared was concern for TAS loss or damage. However, this is not consistent with experimental findings. Of the six published studies that included diagnosed clinically alcohol-dependent individuals (Swift et al. 1992; Sakai et al. 2006; Alessi et al. 2017; 2019; Mun et al. 2021;), none reported TAS loss, damage, or participant challenges. Therefore, perhaps this is not as frequent a challenge as expected. Each alcohol service has a different approach and style of working and so it will be important in the future to interview more staff in a wider range of treatment services. Yet, despite concern for TAS damage, loss and costs, the interview results highlight a willingness of staff to learn and to use TAS with their own service users. Our future parallel publication, will assess the issues of accuracy, feasibility, and acceptability from a service user perspective.

Another common challenge noted by staff was the length of data storage and the potential need for multiple data downloads of the TAS per week. Reflecting on staff answers, we appreciate there will be a clear difference in the utility of TAS when implemented by a research team versus clinical staff. A recommendation for this challenge could be to dedicate someone in a research staff role within the service to address this and include regular data download visits in their responsibility with service users wearing the TAS.

The frequency of research visits for TAS data download has not been noted as a challenge in previous literature, but this may be due to either it being incorporated into the research design, the use of other TAS brands requiring less frequent visits, or the researchers supplying an iPhone to allow participants to conduct their own data download and remove the need for such frequent meetings. No prior study has used clinical staff to implement the TAS wear, all have used researchers, and so this difference is most likely why this has not been noted as a challenge earlier. Practitioner engagement in the treatment has an impact on the service user’s care and engagement (Bright et al. 2017). So, it would be advantageous if staff felt positive and engaged with this technology before implementation. Addressing how often the data needs to be collected will be important for maintaining high ease of use for staff. More extensive work will need to be conducted with staff being responsible for the TAS and visits with service users (rather than a researcher) to assess how they consider TAS ease of use outside a research-guided context.

As mentioned in the above method, the same research team conducted an interview study with service users who wore a TAS for one week and then completed an interview on their experience. Therefore, the views in the paper will be supported or challenged by this service user empirical study in a forthcoming paper.

### Strengths and limitations

A strength of this study is that it is one of the few to include an interview to further explore staff views on TAS. This allowed for exploration and greater detail discussing TAS use, specifically in the context of clinical alcohol treatment. Their insight will be important if TAS are to be implemented into alcohol treatment. Staff knowledge and expertise in implementing and facilitating alcohol treatment and interventions, and experience working with service users, will be required when considering TAS clinical use.

This study is limited by its cohort size and generalizability. We recruited until we reached saturation of interview data but the services that were involved are all based in South London, UK. This meant that no staff from other services were able to participate and include their opinions or experience. We included a range of staff members from each service, but we recognize this is a small sample size of 10. In the future, it would be beneficial to include alcohol services from a wider geographical area. Nevertheless, this is the first study providing evidence of the acceptability and feasibility of TAS from a clinical staff perspective.

Another limitation is that the participant perspectives may be limited given they have not experienced using TAS in practice with their clients. We recognize that this paper reports on staff views on TAS potential rather than used staff experience.

## Conclusion

Our results show that staff are already aware and concerned about the practicalities, acceptability, and feasibility of implementation. Specialist alcohol services have finite budgets and the cost of TAS, not just for the technology but the cost of additional staff meetings, resources, time, and training, is an important consideration. To the best of our knowledge, we conducted the first qualitative interview study on service staff willingness and opinions on TAS. In doing so, we generated new data from a range of South London alcohol service staff that may aid future TAS clinical implementation.

## Data Availability

The data that support the findings of this study are available from the corresponding author, EB, upon reasonable request.

## Abbreviations

DWI: Drinking While Intoxicated
SCRAM: Secure Continuous Remote Alcohol Monitoring
TAC: Transdermal Alcohol Concentration
TAM: Technology Acceptance Model
TAS: Transdermal Alcohol Sensor(s)
UK: United Kingdom
US: United States
